# Large animal *in vivo* evaluation of a binary blend polymer scaffold for skeletal tissue‐engineering strategies; translational issues

**DOI:** 10.1002/term.2007

**Published:** 2015-02-18

**Authors:** James O. Smith, Edward R. Tayton, Ferdous Khan, Alexander Aarvold, Richard B. Cook, Allen Goodship, Mark Bradley, Richard O. C. Oreffo

**Affiliations:** ^1^ Bone and Joint Research Group, Centre for Human Development, Stem Cells and Regeneration University of Southampton UK; ^2^ School of Chemistry University of Edinburgh UK; ^3^ nCATS, Faculty of Engineering and the Environment University of Southampton UK; ^4^ UCL Institute of Orthopaedics and Musculoskeletal Science Royal National Orthopaedic Hospital, Stanmore UK

**Keywords:** bone regeneration, scaffold, skeletal stem cell, binary blend, polymer

## Abstract

Binary blend polymers offer the opportunity to combine different desirable properties into a single scaffold, to enhance function within the field of tissue engineering. Previous *in vitro* and murine *in vivo* analysis identified a polymer blend of poly(l‐lactic acid)–poly(ε‐caprolactone) (PLLA:PCL 20:80) to have characteristics desirable for bone regeneration. Polymer scaffolds in combination with marrow‐derived skeletal stem cells (SSCs) were implanted into mid‐shaft ovine 3.5 cm tibial defects, and indices of bone regeneration were compared to groups implanted with scaffolds alone and with empty defects after 12 weeks, including micro‐CT, mechanical testing and histological analysis. The critical nature of the defect was confirmed via all modalities. Both the scaffold and scaffold/SSC groups showed enhanced quantitative bone regeneration; however, this was only found to be significant in the scaffold/SSCs group (*p =* 0.04) and complete defect bridging was not achieved in any group. The mechanical strength was significantly less than that of contralateral control tibiae (*p <* 0.01) and would not be appropriate for full functional loading in a clinical setting. This study explored the hypothesis that cell therapy would enhance bone formation in a critical‐sized defect compared to scaffold alone, using an external fixation construct, to bridge the scale‐up gap between small animal studies and potential clinical translation. The model has proved a successful critical defect and analytical techniques have been found to be both valid and reproducible. Further work is required with both scaffold production techniques and cellular protocols in order to successfully scale‐up this stem cell/binary blend polymer scaffold. © 2015 The Authors. *Journal of Tissue Engineering and Regenerative Medicine* published by John Wiley & Sons, Ltd.

## 1 Introduction

The application of tissue‐engineering strategies to replace lost skeletal tissue is an area of intense current interest, with significant clinical therapeutic potential. Although many successful laboratory studies have shown potential application for a variety of tissue‐regenerative strategies, there has been limited translation to clinical practice (Mason and Manzotti, 2010). Currently, no synthetic system is able to replicate the diverse biomechanical conditions present within a large organism, so, in order to fully evaluate a candidate biomaterial for skeletal regeneration strategies, a large animal study is required to bridge this translational gap. Despite significant promise, relatively few potential strategies reach scale‐up, primarily due to considerations of cost, regulation, logistics and scale‐up expertise. Additionally, significant findings in small animal models may be harder to reproduce in large animal studies, as a consequence of nutrient/diffusion challenges and comparative scaffold scale‐up that can result in effects that cannot be considered therapeutically beneficial.

Binary‐blend polymers offer the potential to combine different desirable tissue‐engineering properties from two individual polymers, to produce an optimized blend for reparative application. Prior research utilized a combination of *in vitro* tests, as well as a murine femoral segmental defect model, to assess an array of binary‐blend polymers in combination with skeletal stem cells (SSCs) as potential osteogenic bone graft substitutes (Khan *et al.*, 2010). This study highlighted poly(l‐lactic acid)/poly(ε‐caprolactone) (PLLA/PCL), 20/80, as a promising candidate that demonstrated distinct biocompatibility with STRO‐1‐positive, immunoselected SSCs and enhanced bone‐regenerative capacity in a murine study. This polymer blend was therefore chosen as the scaffold for up‐scaling to a large animal long bone segmental defect model, stabilized with a standard configuration external fixator, with the hypothesis that the polymer scaffold would also provide a suitable template for ovine SSC attachment and bone regeneration at a clinically relevant scale. In addition, an aim of this study was to establish a reproducible technique for analysis of candidate biomaterials in a large animal skeletal defect model. Following extensive practical assessments and reviews of other successful large animal skeletal regeneration models (Reichert *et al.*, 2009, 2010; Muschler *et al.*, 2010; Epari *et al.*, 2008; Wallace *et al.*, 1991), we chose to perform this study using a critical defect model in ovine tibiae, secured using a standard configuration rigid external fixator, to control the mechanical environment (Epari *et al.*, 2006; Goodship *et al.*, 1993). Indirect fracture repair is modulated by both biological and mechanical factors; thus, the specific fixator used will influence repair, hence the importance of standardization of the fixator system. We used a defined fixation system, with which we have had previous experience, to control the mechanical influence of the fixation (Goodship, 1992; Kenwright *et al.*, 1991).

## 2 Materials and methods

### 2.1 Polymer scaffold fabrication and preparation

**Figure 1:**
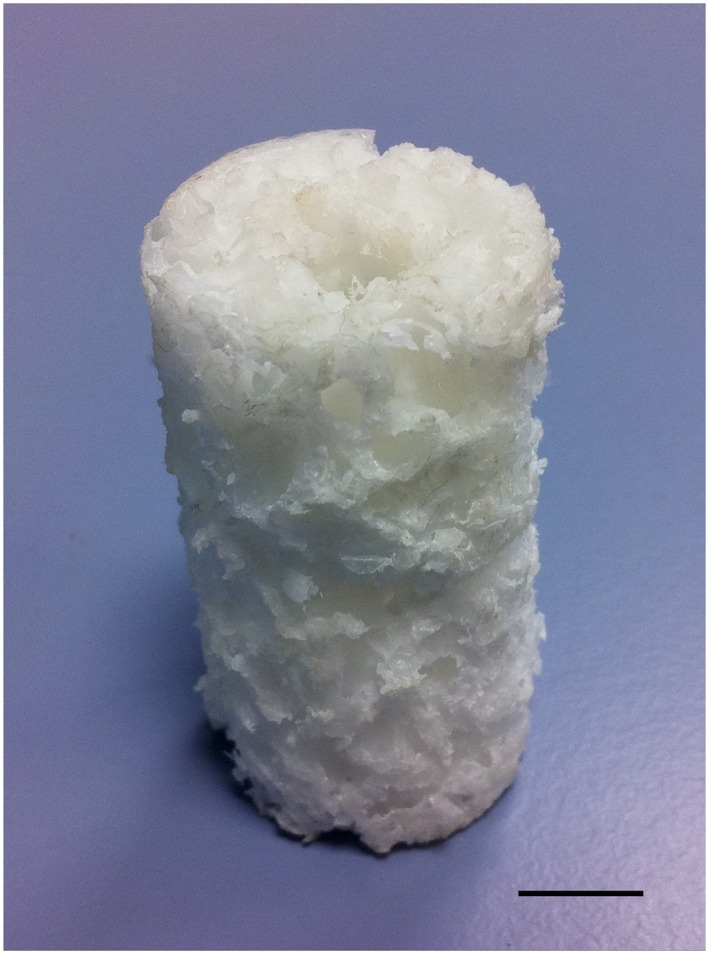
Binary polymer scaffold following processing: note the longitudinal intramedullary canal; scale bar = 10 mm. [Colour figure can be viewed at wileyonlinelibrary.com]

Polymeric binary‐blend PLLA/PCL 20/80 scaffolds, diameter 23 mm, length 35 mm and with an 8 mm longitudinal medullary canal (Figure 1a), were formulated and fabricated using a solution blending process, as previously described (Khan *et al.*, 2010), sterilized by immersion in antibiotic/antimycotic solution (Sigma‐Aldrich, Poole, UK) and degassed using negative pressure. The scaffolds remained in this solution for 24 h before transfer to basal medium [Eagle's minimum essential medium, *α*‐modification (*α*‐MEM) containing 10% fetal calf serum (FCS; Sigma‐Aldrich)] and subsequent UV irradiation overnight.

### 2.2 Preliminary experiments

Scanning electron micrscopy (SEM) was performed on cut sections of scaffold to confirm porosity and define pore sizes. Additionally, a dye‐penetration test was devised to confirm pore interconnectivity: a cuboid of polymer scaffold was prepared (5 × 5 × 40 mm) and supported upright in a specimen pot. The superior aspect was sealed circumferentially using histological embedding wax and Alcian blue dye was infiltrated from the top, so that no dye could pass around the outside of the scaffold. After 12 h, the scaffold below the wax seal was sectioned and examined for the presence of the dye within the pores. Preliminary studies, outside the scope of this study, were performed in order to establish protocols for cell‐seeding densities, scaffold preparation, incubation times and conditions and verification of cell distribution throughout the scaffold. We found that gentle rotation of the scaffold within the culture medium facilitated greater ingress of cells into the scaffold. This was also enhanced by the central longitudinal ‘medullary’ cannulation throughout the scaffold. These measures were employed in the study to maximize an even distribution of cells throughout the scaffold.

### 2.3 Ovine tibial segmental defect model

12 mature ‘cull’ ewe sheep (Northern Mule, weight 60–85 kg) were used for the study, following approval under the Home Office Animals (Scientific Procedures) Act, 1986. All sheep were appropriately assessed, health screened and acclimatized locally for 1 month prior to any intervention; 12 sheep were randomly assigned to one of three treatment groups:

- Group 1 – negative controls (four sheep); empty 35 mm tibial defect.
- Group 2 – positive controls (four sheep); 35 mm tibial defect with polymer scaffold.
- Group 3 – treatment group (four sheep); 35 mm tibial defect with autologous SSC‐seeded polymer scaffold.

#### 2.3.1 Bone marrow harvesting

In order to standardize treatment of the sheep within all groups, bone marrow was aspirated from the iliac crest of all sheep as a separate procedure, approximately 2 weeks prior to the segmental defect operation. Following sedation and anaesthesia, the sheep were placed in right lateral recumbency. Wool was shaved around the left posterior superior iliac crest and the skin was prepared with aqueous iodine scrub. A small incision was made over the aspiration site, and a pre‐heparinized 11‐gauge trocar (Rocket Medical, Watford, UK) inserted into the bone. A pre‐heparinized syringe (0.5 ml, 1000 units/ml) was attached and approximately 5 ml bone marrow aspirated. The aspirate was gently agitated to mix with heparin inside the syringe before transfer to a universal tube and expeditious transport to the laboratory in a cool box.

#### 2.3.2 Isolation, culture and seeding of ovine SSCs

Following aspiration, only marrow from sheep in group 3 underwent isolation, culture and seeding onto the polymer scaffold. Aspirates were centrifuged at 11000 rpm for 4 min and the supernatant removed before resuspending and seeding the cells onto tissue‐culture plastic under osteogenic conditions [basal medium supplemented with 100 μ*m* ascorbate (ascorbic acid 2‐phosphate) and 10 n*m* dexamethasone] at a density of 1 × 10^7^ cells/T175 flask. Medium changes were performed every 3 days until 75–80% confluence had been achieved.

Ovine SSCs were released from monolayer culture and diluted to a concentration of 5 × 10^5^ cells/ml in basal medium. Each group 3 scaffold was seeded by immersion in 20 ml of its respective autologous SSC solution for 2 h, prior to transfer to osteogenic medium (total 1 × 10^7^ cells/scaffold). The seeded scaffolds were then gently rotated under osteogenic conditions for 7 days prior to implantation into the tibial segmental defect model. Scaffolds from Group 2 underwent identical processes, except no cell seeding took place. Prior to implantation, each scaffold was assessed microscopically to confirm absence of infection.

### 2.4 Surgical procedure

#### 2.4.1 *Premedication*, *anaesthesia and preparation*

Sheep were allocated individual pens and food was withheld prior to surgery. A fentanyl transdermal patch (Durogesic, Janssen‐Cilag, High Wycombe, UK) was applied 12 h pre‐operatively to provide analgesia. Prior to anaesthesia, xylazine (Rompun, Bayer Healthcare, Newbury, UK) premedication was administered and each sheep was weighed. Following venous cannulation, anaesthesia was induced using intravenous ketamine (Ketaset, Fort Dodge Animal Health, Southampton, UK) and maintained on inhaled isoflurane (Isoflo, Abbott, Maidenhead, UK). The sheep were intubated with a cuffed endotracheal tube and physiological parameters were monitored. Intravenous antibiotics (Cefalexin, Ceporex, MSD Animal Health, Hoddesdon, Hertfordshire, UK) and maintenance fluids were administered. The sheep were secured in right lateral recumbency and wool was shaved from the entire limb and hindquarter. The skin was prepared with aqueous iodine scrub solution and draped.

#### 2.4.2 ***Operative procedure*** (Figure 2)

**Figure 2:**
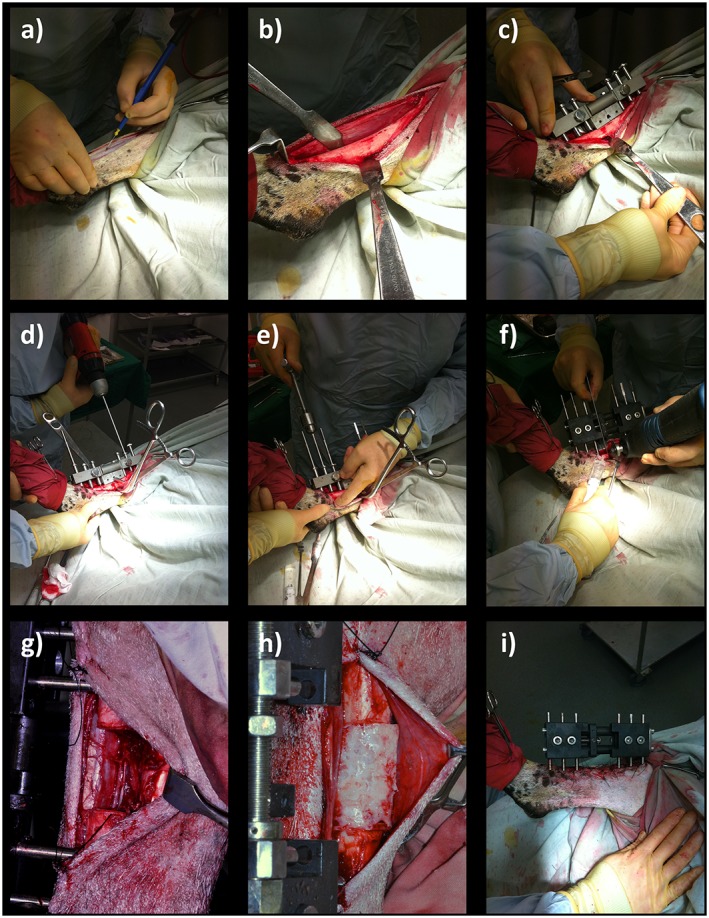
Tibial segmental defect operative procedure. Following skin preparation and draping: (a) an anteromedial approach was used to access the diaphyseal portion of the right tibia; (b) the periosteum was carefully and entirely removed around the site of the proposed ostectomy; (c) the proprietary jig was secured against the bone and the 35 mm ostectomy was marked on the tibia, using diathermy; (d) six 4 mm diameter holes were drilled through the jig guides; and (e) Schanz screws were inserted in a standardized order; (f) the jig was removed and the external fixator was secured in place prior to forming the ostectomy at the pre‐marked site, using an electric reciprocating sagittal saw; (g) the segment of bone was removed, along with any remaining periosteum; (h) polymer scaffold inserted, with or without SSCs; (i) appearance following closure. [Colour figure can be viewed at wileyonlinelibrary.com]

**Figure 3:**
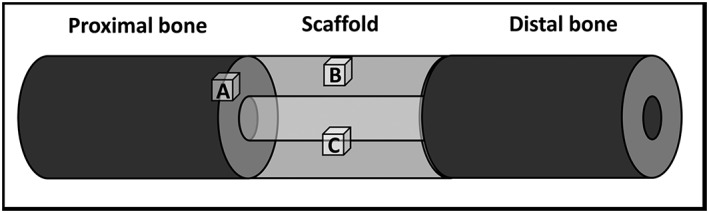
Cubes of tissue/polymer were taken from the indicated regions for histological analysis: (a) the interface between the proximal cut end of tibia and the polymer scaffold; (b) an area on the surface of the mid‐section of the scaffold; (c) an area on the inner face of the scaffold mid‐section

The diaphysis of the right tibia was approached through a craniomedial incision. Periosteum was removed entirely around the site of the proposed ostectomy. The 35 mm ostectomy was marked on the tibia using diathermy. Six 4 mm diameter holes were drilled through the external fixator jig guides and a 6 mm diameter Schanz screw was inserted into each hole by hand, to avoid thermal necrosis or splitting of the bone. Because ovine tibiae widen significantly at the proximal metaphyseal flare, we used two Schanz screws with a 30 mm‐long threaded section proximally and four Schanz screws with a 20 mm‐long threaded section distally. This was replicated for each sheep, and ensured sound bicortical fixation for every screw. The order of insertion of the Schanz screws was also standardized (Table 1). The jig was removed and the custom‐made modular external fixator was secured at a distance of precisely 30 mm from the near tibial cortex to standardize mechanical conditions. The 35 mm ostectomy was made at the pre‐marked site, using an electric reciprocating sagittal saw (Bosch, Uxbridge, UK) with the external fixator in place (three screws proximal, three screws distal). Normal saline was instilled throughout sawing to provide lubrication and prevent thermal necrosis. The segment of bone was removed, along with any remaining periosteum and bone debris, before gentle saline lavage. Animals assigned to groups 2 and 3 (positive controls and treatment group) received the polymer without and with autologous ovine SSCs, respectively (Figure 3). Animals in group 1 (negative controls) remained as empty defects; 5 ml 0.25% bupivacaine (Marcain, AstraZenica, Luton, UK) was infiltrated around the wound for post‐operative analgesia prior to closure. A calibration screw on the external fixator allowed incremental modifications in the defect size, enabling compression fitting of the scaffold between the two cut bone ends. Furthermore, this allowed final adjustments to ensure the ostectomy gap was exactly 35 mm. The fascia was closed with continuous 0 polyglactin (Vicryl, Ethicon) sutures and the skin closed with interrupted 3/0 nylon (Ethilon, Ethicon) mattress sutures. Op‐site spray (Smith & Nephew, Hull, UK) and gauze dressings were applied to the operative site. The entire construct was dressed with wool and crepe bandages and secured using adhesive tape. Craniocaudal and lateral radiographs were made of the entire tibia before anaesthetic reversal.

**Table 1:**
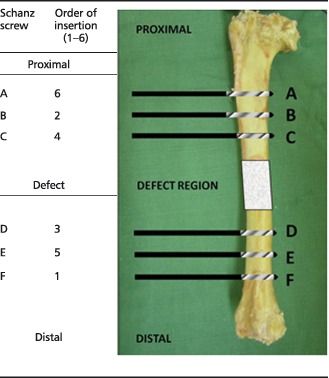
Insertion order of Schanz screws into the ovine tibia

Note that screws at positions A and B had a 30 mm threaded section (20 mm for all other screws).

#### 2.4.4 Post‐operative procedure

Following extubation, the sheep were recovered in individual enclosures. Five cefalexin doses, once daily, were administered and post‐operative analgesia consisted of fentanyl patches for 48 h, followed by buprenorphine (Vetergesic, Alstoe Animal Health, Melton Mowbray, UK) intramuscular injection once daily. The sheep were allowed to mobilize full weight bearing immediately, as tolerated, with unhindered access to food and water for the remainder of the study period. Weekly checks were made of the wounds and fixators to ensure that there was no loosening, and sutures were removed after 14 days.

#### 2.4.5 Post‐operative analysis

Sheep underwent sedation (as previously described) for further radiography of the tibia at 2 and 6 weeks post‐operatively.

### 2.5 Specimen harvest and analysis

Euthanasia was performed 12 weeks after the operative day, using pentobarbital solution 20% (Pharmasol, Andover, UK). The tibiae were disarticulated with fixators *in situ*, the musculature and soft tissue were carefully removed without disturbing the constructs and final radiographs were made. The contralateral tibiae were also harvested and prepared similarly, to act as controls for mechanical testing. The specimens were frozen at –80°C prior to analysis. In addition to post‐operative radiographs, each specimen underwent the following analysis as detailed below.

#### 2.5.1 *μCT analysis*

Samples were scanned using an Xtek Benchtop 160Xi scanner (Xtek Systems, Tring, UK) equipped with a Hamamatsu C7943 X‐ray flat panel sensor (Hamamatsu Photonics, Welwyn Garden City, UK). Scan resolution was up to 31 µm at 150 kV and 60 μA, using a molybdenum target with an exposure time of 534 ms and four‐fold digital gain. Reconstructed volume images were analysed using VGStudio Max 1.2.1 software (Volume Graphics GmbH, Heidelberg, Germany). Initial scans, capturing a 60 mm length centred upon the segmental defect, were used as an overview of new tissue formation. Further high‐resolution scans were made of regions of interest.

#### 2.5.2 Mechanical testing

Following CT visualization, the specimens were defrosted entirely and the constructs were assessed for maximum strength under torque loading. This was deemed the most suitable method, as the mode of failure of long bones of the lower limbs is most frequently through compressive torque. Test constructs were compared with whole tibiae from the contralateral side. The tibiae were potted in quick‐set cement (Polycell, ICI, London, UK) in a custom‐designed rig. The gauge length was measured as the distance between the potted ends of the tibia. The tibiae were tested at a rate of 1°/s in an Instron 8874 (Instron Corp., MA, USA) to 90° of rotation.

Dimensions of the bones were obtained from the intact tibiae. The control tibiae were assumed to be hollow cylinders, with the dimensions based on an average of three measurements of the diameter and wall thickness. Values for the tibiae containing scaffolds were calculated using the scaffold dimensions. The shear modulus (GPa), bone stiffness (Nm/° and Nm/radians), maximum torque (Nm), maximum shear stress (MPa) and maximal angular deformation at failure (°) was calculated for each sample where a definite failure occurred.

#### 2.5.3 Macroscopic analysis

Preparation of the specimens for analysis also enabled a thorough macroscopic evaluation of the integrity of the scaffold material, as well as an assessment of scaffold integration and mode and site of failure.

#### 2.5.4 Histology

Following mechanical testing, several samples, each measuring approximately 10 mm^3^ and representing a region of interest (ROI), were removed from each construct, as follows (Figure 3): (A) the interface between the proximal cut end of tibia and polymer scaffold, to demonstrate any integration that may strengthen the construct; (B) an area on the surface of the mid‐section of the scaffold, to demonstrate any new tissue at the furthest distance from native bone ends, but closely exposed to the host vasculature; (C) an area on the inner face of the scaffold mid‐section, to demonstrate new tissue at the furthest distance from the bone ends, and also distant from the host vasculature, thus relying upon diffusion and new vessel ingrowth for regeneration to take place.

Each ROI specimen was decalcified over a period of approximately 4 weeks, using Tris–EDTA (with Faxitron analysis to confirm complete decalcification), before embedding in wax and cutting into 5 µm semi‐sequential sections and mounting on slides. The slides were stained with Alcian blue and Sirius red (A/S) before visualization using a Zeiss Axiovert 200 inverted microscope (Carl Zeiss, Welwyn Garden City, UK).

#### 2.5.5 Statistical analysis

Sample size calculation was performed using a web‐based program (clincalc.com) with independent study groups, continuous endpoints, *α* error of 0.05 and power of 80%, assuming a change of 15% (±2.5) in control groups. In order to have translational significance, a difference in bone formation of 30% would be required, resulting in a minimum of four/group. This number was used to minimize the use of animals whilst ensuring adequate power for translational statistical significance. GraphPad Prism 6.0 software was used for statistical analysis. Differences between groups were determined using the Kruskal–Wallis test, with *post hoc* Dunn's analysis for non‐parametric data, and were considered to be significantly different at *p <* 0.05.

## 3 Results

### 3.1 Preliminary experiments

**Figure 4:**
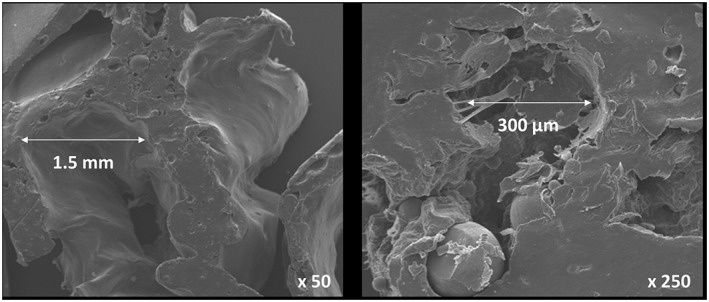
SEM images of polymer scaffold, demonstrating multiple pores of varying dimensions necessary for rapid cellular infiltration

**Figure 5:**
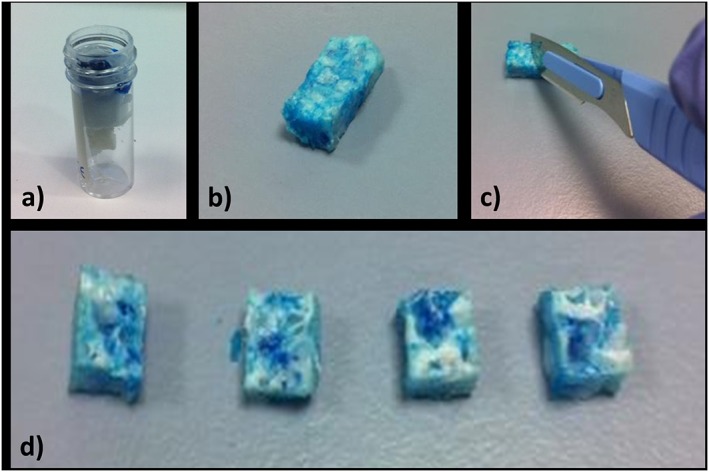
Alcian blue penetration test: (a) the polymer was suspended in a perforated specimen container and the proximal end ‘sealed’ with histology wax before addition of Alcian blue; (b) polymer scaffold following removal of wax – the blue dye is visible throughout the sample; (c) the scaffold was dissected; (d) cross‐sections of the scaffold from proximal (left) to distal (right), demonstrating even penetration of dye throughout the specimen. [Colour figure can be viewed at wileyonlinelibrary.com]

Analysis of the scaffold by SEM confirmed a range of pore diameters of 300 µm–1.5 mm, with significant pore interconnectivity (Figure 4). The Alcian blue penetration test showed considerable permeation of the dye through the scaffold structure, which was confirmed to exist throughout the scaffold by sectional analysis (Figure 5). This further confirmed the pore interconnectivity requisite for diffusion of nutrients and metabolic waste products from cells within the polymer matrix, essential for cellular adhesion, penetration and infiltration.

### 3.2 Ovine tibial segmental defect model

All aspiration, cell‐culture and surgical procedures were technically successful and none of the cell–polymer samples became infected during the *in vitro* stage of the study. One sheep suffered cardio‐respiratory arrest and died shortly following extubation; post‐mortem analysis revealed significant lungworm infestation. Another sheep from the same flock was used to take its place and maintain the original study number of 12 animals. In addition, the external fixator of one sheep (empty defect group) slipped by approximately 1 mm; this was noted on the second post‐operative radiograph. The fixator bolts were re‐tightened immediately *in situ* following recognition of this complication, and no further migration occurred. None of the sheep suffered superficial or deep wound infection, and all ambulated and were fully weight‐bearing at 1 week following operative intervention (Table 2).

**Table 2:** Summary of segmental tibial defect operative procedures in this study, presented in the order of the operative procedure

Study number	Pre‐operative weight (kg)	Operative group	Analysis number	Complications/notes
1	84	Scaffold	5	
2	78	Empty defect	1	
3	75	Scaffold + SSCs	9	
4	85	Scaffold + SSCs	10	
5	75	Scaffold + SSCs	11	
6	65	Empty defect	2	
7	70	Scaffold	6	
8	60	Scaffold	7	
9	75	Scaffold + SSCs	12	
10	60	Scaffold	–	Post‐operative death
11	65	Scaffold	8	
12	75	Empty defect	3	Post‐operative slip of fixation
13	80	Empty defect	4	

Analysis number refers to the number assigned to the specimen for post‐mortem analysis.

### 3.3 Radiographic analysis

**Figure 6:**
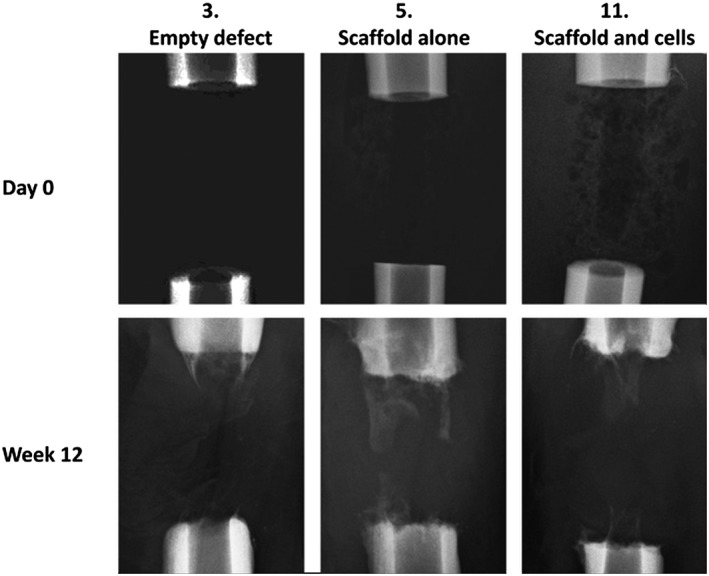
The typical pattern of new bone formation in each group. In the empty defects (3), minimal osteogenesis formed into a conical pattern, mainly from the proximal cortices; in the scaffold‐alone group (5), increased bone formation was evident from both the proximal and distal bone ends, that appeared more evenly throughout the structure of the scaffold polymer; in the scaffold and cells group (11), most bone formation occurred within the central cannulation of the scaffold

Lateral radiographs of each tibia were made incrementally throughout the study (at day 0 and weeks 2, 6 and 12 post‐operatively). No fracture or fixation failure was noted in any group and the fixator stability and defect size was such that union was prevented in all empty defect samples, confirming the generation of a critical‐sized defect (Figure 6). Radiographic analysis, however, showed little osseous formation in any sample of the polymer scaffold groups during the study period. Figure 6 demonstrates the typical pattern of new bone formation in each group: in the empty defect group there was minimal osteogenesis that formed into a conical pattern, predominantly from the proximal cortices; in the scaffold‐alone group, increased bone formation was evident from both the proximal and distal bone ends, that appeared more evenly throughout the structure of the scaffold polymer; in the scaffold and cells group, most bone formation occurred within the central cannulation of the scaffold. Although minor areas of calcified tissue are seen projecting particularly from the proximal bone ends in all groups, union was not demonstrated in any specimen after 12 weeks.

### 3.4 Micro‐computed tomography (μCT) analysis

**Figure 7:**
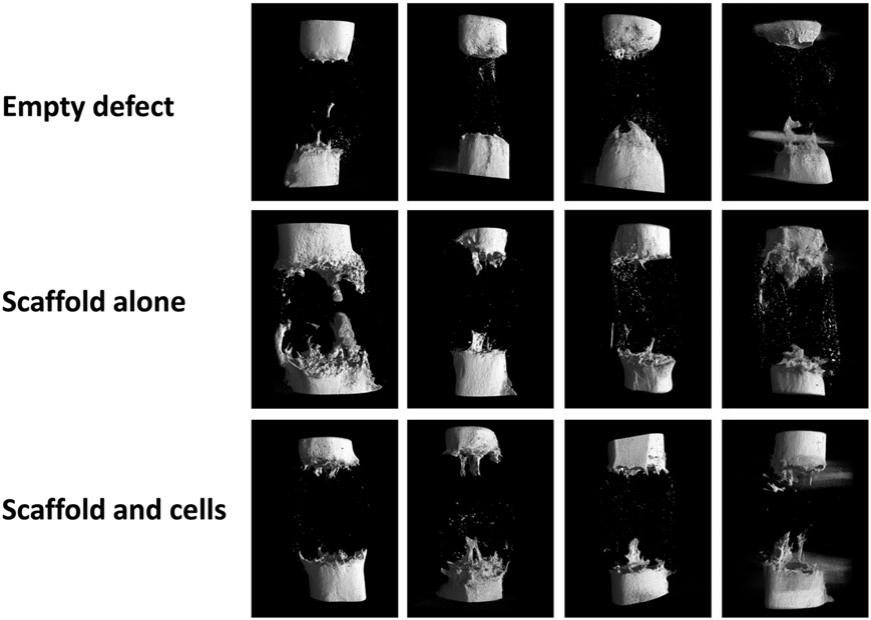
Quantitative μCT analysis at 12 weeks post‐operation: there is minimal regenerative activity in the empty defect specimens; some bone formation is seen in both scaffold groups, although there is no certain difference in the group with added SSCs

Analysis using quantitative CT radiography produced reformatted images of each specimen centred upon the defect site (Figure 7). These images largely confirmed the plain radiographic findings, that empty defects undergo a process of atrophic non‐union. There was little difference in bone formation between the two scaffold groups; however, more regeneration was confirmed in these latter groups compared to empty‐defect controls.

**Figure 8:**
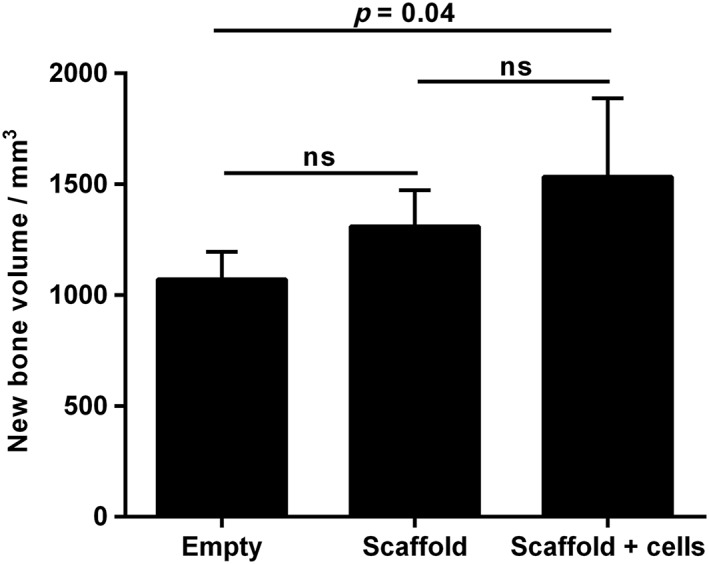
Quantitative μCT analysis of new bone formation after 12 weeks of incubation; error bars, SD; ns, not significant

Quantitative analysis of the volume of new bone formation within the osteotomy site revealed a trend towards increasing bone formation in the scaffold alone and scaffold with SSCs groups when compared to the empty‐defect group, which reached statistical significance (*p =* 0.04 for empty vs scaffold with SSCs) (Figure 8).

### 3.5 Mechanical testing

**Figure 9:**
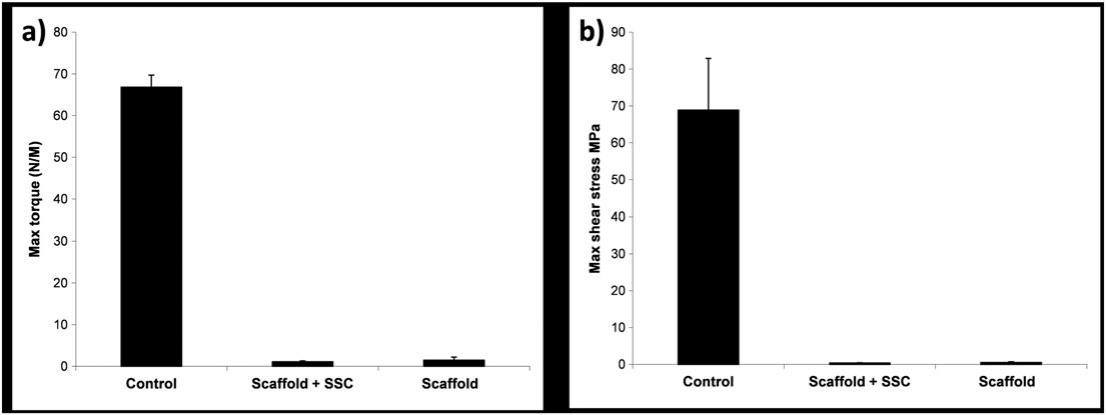
Results of mechanical testing for the sheep tibiae under torsional compression, demonstrating: (a) maximum torque; and (b) maximum shear stress before failure; control refers to the contralateral intact tibia; error bars, SD

Bones were tested to failure at 1°/s. Only samples which demonstrated a definite failure before 40° were included in the analysis, to ensure that accurate values could be ascribed to a specific point of failure. This excluded all the empty‐defect samples and one of the scaffold‐containing samples. The mean shear modulus for intact ovine tibiae was 2.45 GPa (SD 0.73) and the mean maximum torque was 66.82 Nm (SD 2.88). All the tibiae with defects were significantly impaired mechanically compared to the contralateral control (Figure 9). Tibiae from the negative control group failed to provide a detectable failure, as did one of the positive controls. There was no significant difference in the mechanical properties of the positive controls and the treatment groups.

### 3.6 Macroscopic analysis

**Figure 10:**
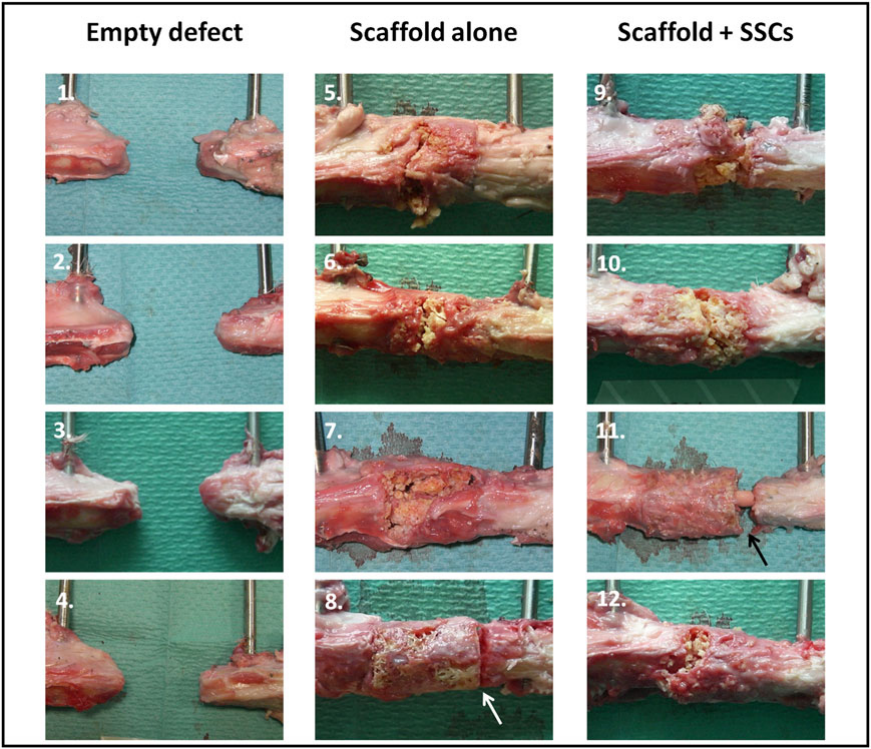
Macroscopic specimen analysis following mechanical testing. Regions of interest in each image show the proximal diaphysis (left), the distal diaphysis (right) and defect containing no scaffold (1–4), scaffold alone (5–8) or scaffold with cells (9–12). Failure occurred through the scaffold itself in three of the four specimens in each scaffold group; however, failure occurred at the distal scaffold–diaphysis interface in one specimen of each scaffold group (arrows in 8 and 11). [Colour figure can be viewed at wileyonlinelibrary.com]

Following mechanical testing, macroscopic analysis of each specimen (Figure 10) confirmed that atrophic non‐union had occurred in every empty‐defect specimen. As demonstrated by plain radiographic and μCT analysis, minimal osteogenesis was observed in these specimens, forming a conical projection mainly from the proximal cortices. In the scaffold‐alone group, increased bone formation was evident from both the proximal and distal bone ends, obscuring the interface between the bone and polymer scaffold, except in one case (white arrow in Figure 10). The interface between scaffold and diaphysis appeared considerably more indistinct in the scaffold + SSCs group, with coverage of the scaffold by soft reparative tissue in continuity with bone. In three of the four specimens in both scaffold groups, failure occurred through the scaffold itself, usually as a transverse or short oblique fracture line. Failure occurred at the distal scaffold–diaphysis interface in one specimen of each scaffold group (white and black arrows, Figure 10, insets 8 and 11).

**Figure 11:**
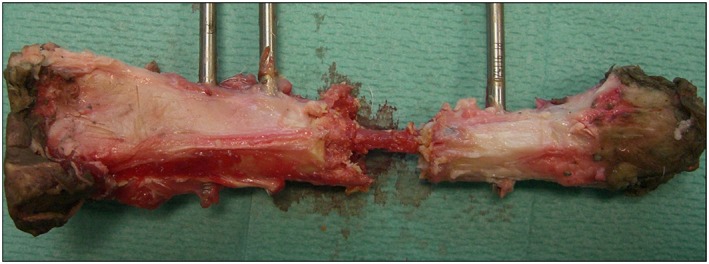
Macroscopic image of a tibia treated in the scaffold + SSCs group (specimen 9 in Figure 10). The polymer scaffold in this case was largely fragmented following mechanical testing and has been carefully removed to reveal a central bridge of new tissue formation within the medullary cavity of the scaffold; note full continuity between the proximal and distal diaphyseal segments. [Colour figure can be viewed at wileyonlinelibrary.com]

A feature of new tissue growth that was seen only in specimens from the scaffold + SSCs group was intramedullary growth within the central canal of the polymer scaffold (Figure 10, inset 11, and Figure 11). The extent of this tissue growth was not appreciated by radiographic imaging modalities, as the tissue was not fully calcified and had a similar density to that of the surrounding polymer scaffold; however, it provided full continuity between the proximal and distal bone segments in these specimens.

### 3.7 Histological assessment

**Figure 12:**
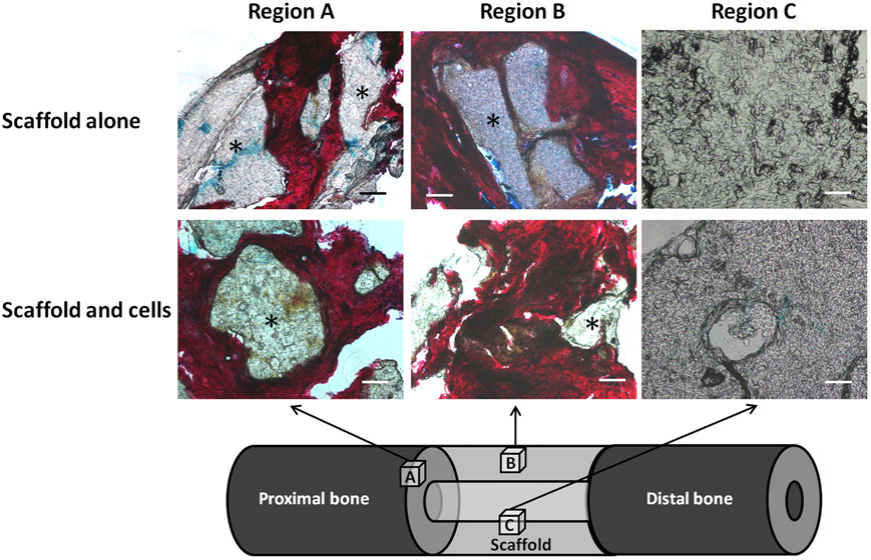
A/S histological analysis of the ovine segmental tibial defect model after 12 weeks *in vivo* incubation. Only the scaffold groups are displayed, as insufficient tissue was formed within the empty defects. In region A (bone–scaffold interface), there was significant infiltration of new tissue into the scaffold, as demonstrated by red staining of collagen type I. In region B (near the surface of the mid‐section of the scaffold), new osseous tissue was seen in both scaffold groups, with deep penetration of tissue into the porous network. Region C (within the inner face of the scaffold mid‐section) showed no new osseous tissue formation in either scaffold group; the polymer in this region remained intact, with some surrounding cells but no new bone formation; *polymer; scale bars = 100 µm. [Colour figure can be viewed at wileyonlinelibrary.com]

Histological examination of the three ROIs revealed a consistent pattern of tissue regeneration within the defect site in both groups containing polymer scaffold (Figure 12). Insufficient tissue was formed within the empty‐defect group to enable histological processing and analysis. In both the scaffold‐alone and scaffold with SSCs specimens, there was significant infiltration of new tissue into the polymer scaffold at the bone–scaffold interface (region A), as demonstrated by staining of collagen type I with Sirius red. In this zone, most pores within the scaffold had been filled by new bone and entirely surrounded the polymer ‘islands’; cells and matrix were seen within the pores of the scaffold and appeared to surround the fragmented polymer. In region B, near the surface of the mid‐section of the scaffold, new osseous tissue was consistently seen in both scaffold groups, with direct contact of this red‐staining tissue with the polymer and deep penetration of tissue and cells into the porous network. There was no appreciable difference in cell number or concentration between the scaffold groups in regions A or B and only very limited and sporadic Alcian blue staining in both regions. Region C, within the inner face of the scaffold mid‐section, showed no new osseous tissue formation in either scaffold group. The polymer in this region remained intact, with some surrounding cells but no new bone formation.

## 4 Discussion

The purpose of this study was to assess the efficacy of a binary‐blend polymeric scaffold (PLLA/PCL, 20/80) that had already proved successful *in vitro* and in a small animal *in vivo* model (Khan *et al.*, 2010), and to establish an appropriate large animal critical defect model that could be used consistently for the evaluation of putative tissue‐engineering constructs for skeletal regeneration. Our scaffold was designed to exhibit good porous interconnectivity and a variable pore size, to optimize mass transport, cell migration and mechanical integrity. Initial studies proposed 100–300 µm as the optimal pore diameter range for osteogenesis; however, the requirement of large pores for vascular invasion and pore interconnectivity for enhanced bone formation have more recently been appreciated and the exact size of pores may not, in itself, have a critical influence on bone regeneration *in vivo* (Klawitter *et al.*, 1976; Murphy *et al.*, 2010; Williams *et al.*, 2005; Roosa *et al.*, 2010). Following preliminary research and review to define the most appropriate animal and anatomical site for testing, an ovine tibial segmental defect model with external fixation was chosen. Sheep are readily available, economical and docile. In addition, sheep share important similarities to humans (similar body weight and bone dimensions), which make them useful as test subjects for musculoskeletal investigations (Newman *et al.*, 1995; Ravaglioli *et al.*, 1996; Taylor *et al.*, 2006). Furthermore, ovine bone mineral composition, metabolic and remodelling rates are roughly equivalent to those of humans; however, some researchers maintain that significant histological differences exist, particularly in younger sheep (<7 years) prior to secondary osteonal remodelling (Reichert *et al.*, 2010). The differentiation potential of ovine‐derived SSCs has also recently been characterized, enabling laboratory protocols to be defined to induce osteogenic differentiation in ovine cells (McCarty *et al.*, 2009). External fixators are particularly appropriate to this study, as they are used clinically in bone defect surgery and, critically, external fixators offer additional advantages during experimental procedures, as they do not affect the defect site and allow easier radiographic and histological analysis, whilst precisely standardizing the mechanical stability of the defect (Goodship *et al.*, 1993; Gugala *et al.*, 2007).

Aspiration, storage and processing of ovine bone marrow aspirate was successful and *in vitro* growth of ovine SSCs on the polymer was rapid and appeared to penetrate deeply and uniformly within the structure of the scaffold, without observed cellular necrosis. Thus, an appropriate ovine cell collection, seeding and *in vitro* incubation protocol has been defined for application of large polymeric scaffolds to bone defects, and this could be readily replicated in future similar studies.

Most aspects of the segmental defect operative procedure itself were also successful. Apart from an explained death following general anaesthesia, and a single incidence of minor fixator slippage which was easily rectified, all aspects of sheep handling, premedication, anaesthesia, aspiration, preparation, ostectomy, fixation, recovery, analgesia, post‐operative care, radiography, termination and sample harvesting proceeded without incident. Furthermore, due to the lack of bone formation in the empty‐control groups, this study has confirmed the critical nature of the 3.5 cm mid‐shaft tibial defect, hence making it an appropriate model for future construct analysis. The experimental period of 12 weeks was chosen to be applicable to clinical fracture healing.

Several aspects of post‐mortem analysis required modification to account for the large specimens resulting from the study. Consequently, some of the analysis was rationalized to allow processing – histological preparation, in particular, was limited by a requirement to decalcify all tissue prior to processing, as well as difficulties in preparing and cutting polymeric constructs. Therefore, only small regions could be analysed separately, and limited numbers of slides were available for staining. Plain radiographic and μCT results correlated closely; thus, the model has been shown to create a replicable critical‐sized segmental defect using all analytical modalities. Addition of the scaffold and cells significantly enhanced bone formation when compared to the empty defect, although this did not reach statistical significance with the addition of scaffold alone. Furthermore, soft tissue formation appeared to be enhanced by the addition of SSCs to the construct, as visualized macroscopically and on histological analysis. This may indicate a prequel to eventual osteogenesis and, although the time periods involved for this model to demonstrate complete union are too long to be clinically favourable, they do provide a starting point for future studies based on this ovine model and related polymer‐blending technology, underlining the importance of SSCs as part of this approach. Even though modest new calcified bone was seen on radiographic analysis, the histological results demonstrate regenerative tissue forming throughout the circumference of the scaffold and penetrating to some extent into the porous scaffold substance. However, no new tissue was seen within the central scaffold area. This area would be expected to regenerate last because it is located deep within the polymer, furthest from osteogenic influences of the bone ends and periosteum and poorly exposed to the surrounding vasculature. Tissue that was formed stained uniformly with Sirius red, indicating a high proportion of type I collagen. Conversely, modest Alcian blue staining was seen, confirming osteogenic rather than chondrogenic differentiation, appropriate to a skeletal regeneration strategy. The relative absence of Alcian blue staining is suggestive of intramembranous bone formation and contrasts with the results from murine femoral defect studies performed by our group, in which significant areas of chondrocytic activity were noted, consistent with endochondral ossification (Khan *et al.*, 2010, 2013). These studies utilized less rigid intramedullary fixation of the osteotomy sites, rather than the relatively stable external fixator construct used in the present study. Future work could be directed at modulating the mechanical environment at the osteotomy site by changing fixator stability to influence the type of bone formation. The presence of abundant uncalcified matrix suggests that relatively small changes in the protocol may lead to substantial bone development.

Mechanical strength testing confirmed that the contralateral (unoperated) tibiae possessed strength characteristics similar to previously published results for mammalian bone: shear moduli for human and bovine cortical bone have been published as 3.51 and 4.14 GPa, respectively (Cowin, 1989), which correspond with the mean ovine tibial values of 2.45 GPa (SD 0.73) recorded. The mean maximum torque for ovine tibiae in the current study was 66.82 Nm (SD 2.88), which correlates closely with previously published ovine tibia destructive tests (Jamsa and Jalovaara, 1996). Additionally, torsional stiffness values are similar to those previously published for ovine tibiae (Reichel *et al.*, 1998; Gao *et al.*, 1997). These studies validate the current methods for mechanical strength testing and justify the assumption required for calculation, that the tibiae are cylindrical. The findings that all mechanical strength modalities tested were significantly lower for the test tibiae, and that no significant increase was obtained by the addition of scaffold or scaffold and SSCs is therefore valid.

All the analyses confirmed a trend towards increasing bone formation with the polymer scaffold when compared to the empty defect, although this effect has not been proved to be significant. Furthermore, the addition of autologous SSCs, at the dose used, to the construct appears to further enhance skeletal regeneration and may accelerate the formation of precursor tissue. We can therefore conclude that the presence of SSCs was an important and necessary factor for the enhancement of tissue formation seen, although the interaction between the SSCs and the scaffold itself, within the tissue‐engineering approach, was clearly important. Multiple additional factors must be considered when up‐scaling a successful small animal study and each may account for the failure of additional regeneration seen in this study (Table 3). These reasons highlight some of the hurdles that need to be overcome before successful outcomes can be expected from large animal trials in our laboratories, and may be as diverse as avoiding local anaesthetic use around the operative site, to defining new techniques to limit thermal necrosis (Kuttenberger *et al.*, 2010; Tayton *et al.*, 2012). This also serves to underline the critical importance of appropriate large animal studies in the assessment of therapeutic products, so that any adverse factors can be resolved prior to human clinical application.

**Table 3:** Factors to consider and modify prior to further large animal skeletal engineering biomaterial evaluation

Initial *in vitro* scaffold incubation
Scaffold porosity and pore interconnectivity
Absence of impermeable scaffold surface coating
Dynamic cell seeding and incubation; consider using a bioreactor
Confirmation of cell viability and scaffold penetration prior to implantation
Reduce transit time prior to implantation
*In vivo* incubation
Size of segmental defect
Operative technique; removal of periosteum
Potential toxicity of operative adjuncts, e.g. local anaesthetic infiltration
Construct stiffness, stability and micromotion
Duration of incubation
Analytical factors
Requirement for decalcification prior to analysis
Ability to analyse large specimens for microscopic and macroscopic signs of integration
Ability to differentiate between scaffold and new tissue growth

A potential weakness of our study is the small size (*n* = 4/group). This was based upon a power calculation that assumed a difference between test and control bone formation of 30% to have translational validity. Although we did not see such increased bone formation in this study, several factors support the use of low numbers for this research. Part of the study aim was to establish a consistent and reproducible technique for stabilization of a long bone critical defect in a large animal, suitable for analysis and trialling of tissue‐regeneration strategies. This was achieved. An additional objective was to use this model to test a specific tissue‐engineering construct for bone regeneration. We have been able to show that this construct does not fulfil all the criteria for direct translation to clinical trials whilst minimizing the number of animals needed and costs involved. It is envisaged that, using the experience gained from this study, we will be able to use the same model with few refinements to test alternative strategies in future. Tissue engineering is difficult – it requires the coordinated interplay of a range of agencies with a wealth of resources and knowledge for success. This study adds to that knowledge base and allows further incremental changes to be made to approach a valid clinical strategy.

In conclusion, a consistent tibial critical defect model in sheep has been established; however, significant additional work is required to establish working protocols and improve the tissue‐engineered constructs to aid scale‐up and subsequent generation of tissue for skeletal repair.

## Conflict of Interest

The authors declare no conflicts of interest.
